# CCIBP: a comprehensive cosmetic ingredients bioinformatics platform

**DOI:** 10.1093/bioinformatics/btad416

**Published:** 2023-07-03

**Authors:** Linlin Gong, Rui Zhang, Mengying Han, Qian-Nan Hu

**Affiliations:** CAS Key Laboratory of Computational Biology, CAS-MPG Partner Institute for Computational Biology, Shanghai Institute of Nutrition and Health, Shanghai Institutes for Biological Sciences, University of Chinese Academy of Sciences, Chinese Academy of Sciences, Shanghai 200031, P.R. China; CAS Key Laboratory of Computational Biology, CAS-MPG Partner Institute for Computational Biology, Shanghai Institute of Nutrition and Health, Shanghai Institutes for Biological Sciences, University of Chinese Academy of Sciences, Chinese Academy of Sciences, Shanghai 200031, P.R. China; CAS Key Laboratory of Computational Biology, CAS-MPG Partner Institute for Computational Biology, Shanghai Institute of Nutrition and Health, Shanghai Institutes for Biological Sciences, University of Chinese Academy of Sciences, Chinese Academy of Sciences, Shanghai 200031, P.R. China; CAS Key Laboratory of Computational Biology, CAS-MPG Partner Institute for Computational Biology, Shanghai Institute of Nutrition and Health, Shanghai Institutes for Biological Sciences, University of Chinese Academy of Sciences, Chinese Academy of Sciences, Shanghai 200031, P.R. China

## Abstract

**Summary:**

Cosmetics form an important part of our daily lives, and it is therefore important to understand the basic physicochemical properties, metabolic pathways, and toxicological and safe concentrations of these cosmetics molecules. Therefore, comprehensive cosmetic ingredients bioinformatics platform (CCIBP) was developed here, which is a unique comprehensive cosmetic database providing information on regulations, physicochemical properties, and human metabolic pathways for cosmetic molecules from major regions of the world, whilst also correlating plant information in natural products. CCIBP supports formulation analysis, efficacy component analysis, and also combines knowledge of synthetic biology to facilitate access to natural molecules and biosynthetic production. CCIBP, empowered with chemoinformatics, bioinformatics, and synthetic biology data and tools, presents a very helpful platform for cosmetic research and development of ingredients.

**Availability and implementation:**

CCIBP is available at: http://design.rxnfinder.org/cosing/

## 1 Introduction

“Cosmetics” is defined as “a product (excluding pure soap) intended to be applied to the human body by smearing, spraying, misting, or other means for the purpose of cleaning, beautifying, enhancing glamour, or altering the appearance” (Cosmetics & U.S. Law | FDA, https://www.fda.gov/cosmetics/cosmetics-laws-regulations/cosmetics-us-law, 2023). For thousands of years, people have used cosmetics to enhance their appearance and cleanse their bodies, with the cosmetics industry being expected to be worth $75.84 billion by 2025. In China, cosmetics consumed by people account for 0.91% of the total retail sales of social consumer goods, and this is continuing to increase annually (Cosmetic market value worldwide, 2018–2025 | Statista, https://www.statista.com/statistics/585522/global-value-cosmetics-market/, 2023).

With the improvement in people’s requirements for quality of life and skin management, consumers’ cognition of cosmetic ingredients continues to improve. People explored the functional ingredients and product formulations in cosmetics to choose the most suitable cosmetic products for their skin conditions. In today’s carbon-neutral environment, the concept of pure beauty has risen rapidly. People tend to choose safer natural products, mainly showing natural, mild, green, and environmentally friendly flowers, trees, and traditional Chinese medicinal ingredients. For cosmetics companies, in the face of the growing demand for beauty cosmetics, large-scale industrial production of certain ingredients is required to obtain the minimum production costs and maximum profit.

However, some of the current cosmetics databases are regional, whilst some are commercial, such as CIR (Cosmetic Ingredient Review, http://www.cir-safety.org/), CTFA (Cosmetic, Toiletry & Fragrance Association, https://www.ctpa.org.uk/), Kosmet (https://www.kosmet.com/), and ChinaCosIng (https://www.chinacosing.com), and their content is mainly focused on market and commercial information. Therefore, comprehensive cosmetic ingredient bioinformatics platform (CCIBP) was developed, which is the first comprehensive cosmetic ingredient database, containing basic cosmetic safety regulations and cosmetic ingredient information for major regions of the world. Additionally, it manually sorts the nine most popular functional ingredient molecules, whilst containing the natural plant information corresponding to the molecule and its traditional Chinese medicine information. Finally, knowledge on synthetic biology is integrated to recommend possible biosynthetic pathways. This can not only provide consumers with simple and clear ingredient information, but aid merchants in mining new ingredients and developing new products.

## 2 Materials and methods

### 2.1 Data collection

**Figure 1.**
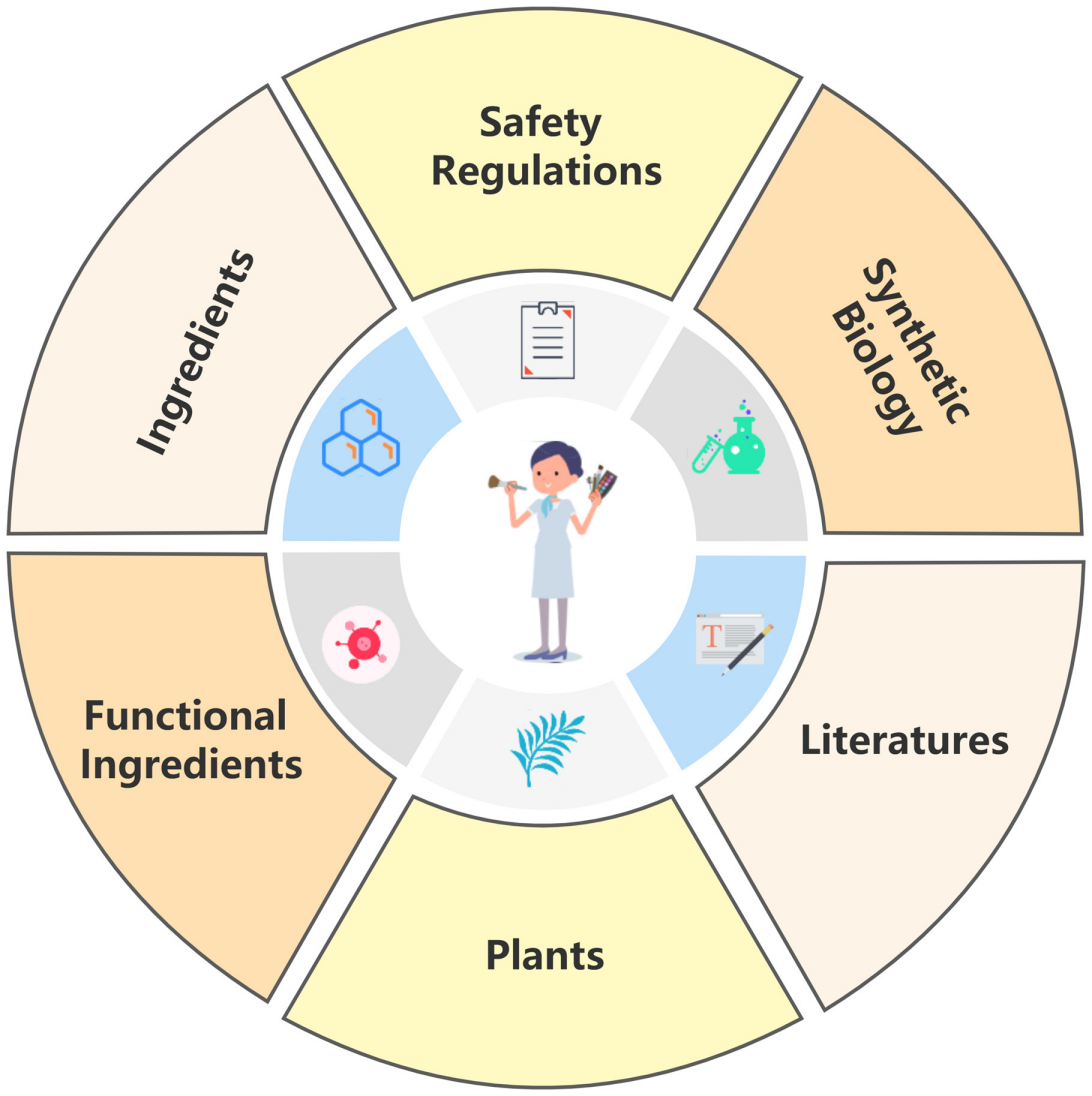
Introduction to the structure of CCIBP

The cosmetics ingredients regulations mainly come from relevant safety regulations and some authoritative associations in various countries and regions. Human metabolic pathway information was obtained from the HMDB (Wishart *et al.* 2022) database, while Plant data were obtained from CMAUP (Zeng *et al.* 2019) and the Flora of China *et al.* The cosmetic detailed ingredients information was obtained from the China National Medical Products Administration. Uniquely, cosmetic ingredients were empowered by synthetic biological reactions and pathways manually curated from the literature (Fig. 1).

### 2.2 Database contents

The database primarily contained five data types:

#### 2.2.1 Safety regulations

For cosmetic products, safety is the most important factor, therefore we have collected the laws and regulations on cosmetics in the United States, the European Union, China, and other regions, and statistics on various types of cosmetic molecules that are clearly stated, primarily including the cosmetic ingredient catalog, prohibited molecules, restricted molecules, sunscreens, hair dyes, preservatives, and colorants (Mishra *et al.* 2022).

#### 2.2.2 Cosmetic ingredients information

The basic physical and chemical information for each cosmetic molecule are provided, which users can obtain by simply entering the molecule name. Natural language processing technology was used to investigate the relationships between compounds, genes, and species from a large number of studies to provide scientific references (Kaushik *et al.* 2020).

#### 2.2.3 Relevant plants information

Natural ingredients in cosmetics are highly sought after by users and merchants, among which plants are the most common, with many products using extracts from plant roots, leaves, flowers, fruits, and other parts. Therefore, we associated the molecule with its plants (Lei *et al.* 2022) and provided detailed plant taxonomic information, among which we also collected information on traditional Chinese plants, particularly information on traditional Chinese medicine, which is very helpful in understanding the pharmacology of molecules.

#### 2.2.4 Functional molecules and their formulations

Efficacious molecules play a decisive role in cosmetics. When designing product formulations, the efficacy system is the key point of innovation and the selling point, and it is the first step that needs to be determined, whereas other system designs must be based on the basic principle of assisting and enhancing the efficacy system. Therefore, we counted 1980 products for the nine most popular types of efficacy molecules, analyzed their formulas and the use of efficacy molecules, and provided references for formula design. Simultaneously, we can provide users with concise and quick product formula molecular information.

#### 2.2.5 Synthetic biological reactions and pathways

Compounds obtained by microbial or enzymatic processes can be classified as “natural” under US and European regulations, so using a biocatalytic route allows cosmetic companies to declare that most of their ingredients are of natural origin. This includes synthetic biology and fermentation engineering strategies, in line with the green and sustainable concept; therefore, this was also studied here. We use our method RxnFinder (Hu *et al.* 2011) to calculate the molecules similar to the target molecule and sort them according to the similarity percentage, whilst also providing the basic physical and chemical information of these similar molecules and their participation in human metabolism related information. Additionally, we also used synthetic biology knowledge and computer technology to provide possible biosynthetic pathways for target molecules, which is of great significance for the large-scale industrial production of cosmetic ingredients, in line with the expectations of merchants to reduce costs and users’ green safety.

## 3 Conclusions

CCIBP is a unique comprehensive online cosmetic database of cosmetic molecules, including safety regulations, human metabolic pathways, plant information on natural molecules, and efficacy molecules. It provides consumers with concise and comprehensive molecular information about cosmetics whilst being clear and friendly to ordinary users. This can supplement users with beauty knowledge from a professional perspective to choose the most suitable beauty products for them.

Additionally, it also provides an important reference for cosmetic companies to design efficacy systems and product formulations and gives legal guidance for the internationalization of products. At the same time, it includes knowledge of synthetic biology, which is of great significance for the development of new ingredients.
